# Chronobiology of the Tumor Microenvironment: Implications for Therapeutic Strategies and Circadian-Based Interventions

**DOI:** 10.14336/AD.2024.0327

**Published:** 2024-03-27

**Authors:** Dengxiong Li, Qingxin Yu, Ruicheng Wu, Zhouting Tuo, Weizhen Zhu, Jie Wang, Fanglin Shao, Luxia Ye, Xing Ye, Koo Han Yoo, Mang Ke, Yubo Yang, Wuran Wei, Dechao Feng

**Affiliations:** ^1^Department of Urology, Institute of Urology, West China Hospital, Sichuan University, Chengdu, China.; ^2^Department of pathology, Ningbo Clinical Pathology Diagnosis Center, Ningbo, Zhejiang, China.; ^3^Department of Urology, The Second Affiliated Hospital of Anhui Medical University, Hefei, China.; ^4^Department of Rehabilitation, The Affiliated Hospital of Southwest Medical University, Luzhou, China.; ^5^Department of Public Research Platform, Taizhou Hospital of Zhejiang Province Affiliated to Wenzhou Medical University, Linhai, China.; ^6^Cedars-Sinai Medical Center, Los Angeles, CA 90048, USA.; ^7^Department of Urology, Kyung Hee University, Korea.; ^8^Department of Urology, Taizhou Hospital of Zhejiang Province Affiliated to Wenzhou Medical University, Taizhou, China.; ^9^Department of Urology, Three Gorges Hospital, Chongqing University, Wanzhou, Chongqing, China.; ^10^Division of Surgery & Interventional Science, University College London, London W1W 7TS, UK.

**Keywords:** circadian rhythm, tumor microenvironment, tumor-infiltrating cells, chronotherapy

## Abstract

Numerous research works have emphasized the critical role that circadian rhythm plays in the tumor microenvironment (TME). The goal of clarifying chrono-pharmacological strategies for improving cancer treatment in clinical settings is a continuous endeavor. Consequently, to enhance the use of time-based pharmaceutical therapies in oncology, combining existing knowledge on circadian rhythms' roles within the TME is essential. This perspective elucidates the functions of circadian rhythms in the TME across various stages of cancer development, progression, and metastasis. Specifically, aging, angiogenesis, and inflammation are implicated in modulating circadian rhythm within the TME. Furthermore, circadian rhythm exerts a profound influence on current cancer treatments and thereby generates chronotheray to manage tumors. From a TME perspective, circadian rhythm offers promising opportunities for cancer prevention and treatment; nevertheless, further study is needed to address unanswered scientific problems.

## 1. Introduction

The circadian rhythm, or circadian clock, is integral to human life, influencing activities within 24-hour cycles by regulating over half of protein-coding gene expressions [1, 2]. A well-functioning circadian rhythm can reduce the risk of numerous diseases, such as type 2 diabetes and Alzheimer’s [3]. Conversely, circadian disruptions can impair regulatory networks, potentially leading to various illnesses, including metabolic syndrome, sleep disorders, sex function, and notably, carcinogenesis [4, 5]. Contemporary work demands often necessitate nocturnal working hours, thereby disturbing normal circadian rhythm and consequently escalating cancer incidences [6]. Additionally, modern lifestyle changes, such as nocturnal leisure activities, further contribute to circadian disturbances [7, 8]. These disruptions can foster cancer initiation through mechanisms such as metabolic disorders, DNA damage, and cellular senescence [9]. Furthermore, circadian disruptions influence cancer progression by modulating inflammatory responses, stem cell growth, and treatment efficacy [10-12]. For example, in gastric cancer, modulating the expression of clock genes reversed trastuzumab resistance [13]. In clinical practice, the timing of therapy can significantly enhance the effectiveness of cancer treatments [14, 15]. Circadian clocks are pivotal in regulating and maintaining the tumor microenvironment (TME), a complex network comprised of various cellular and non-cellular components instrumental to cancer initiation and progression [16]. A series of researches have centered on the interplay between circadian rhythm and the TME, particularly in the regulation of cancer stem cells within the TME, ultimately impacting the efficacy of cancer therapies [17]. While a few studies have attempted to delineate the role of circadian clocks in the TME and their implications for cancer treatments [18, 19], these investigations are somewhat limited due to a dearth of original findings. In light of recent researches that have shed light on the relationship between circadian rhythm and the TME, we undertook a comprehensive review of current literatures, thereby encapsulating the integral function of circadian rhythm within the TME and their potential to inform cancer management strategies.

## 2. Circadian rhythm and its role in TME during carcinogenesis

Circadian disruption, often resulting from night shift work schedules, is a recognized facilitator of the carcinogenesis process [9]. The tissue microenvironment plays a crucial role in this regard. For instance, in colitis tissue, the knockout of BMAL1 in PD-L1+ regulatory B cells inhibited the transcription of IL-33, resulting in the death of CD4+ T cells. This consequent reduction of CD4+ T cells in colitis tissue is postulated to heighten the risk of colitis-associated colorectal cancer [20]. In a separate study, the ablation of BMAL1 expression in intestinal epithelium was correlated with an increased initiation of cancer in mouse intestinal organoids [21]. Mechanistically, the loss of BMAL1 expression or circadian disruption can suppress the Wingless and Int-1 pathways. Furthermore, the Hippo pathway may be activated following the loss of BMAL1 expression or circadian disruption in intestinal epithelium. These two studies collectively imply that circadian disruption facilitates colon tumor initiation by modulating clock-relative genes both in the immune and epithelial cells within the tissue microenvironment. In addition to protein-coding genes, lncRNAs are also involved in tumorigenesis through circadian disruption. For instance, ADIRF-AS1, a downstream lncRNA of the BMAL1/ CLOCK complex, promoted renal carcinogenesis by suppressing the transcription of the PBRM1/BRG1 complex in mouse models [22]. Moreover, ADIRF-AS1 also regulates the extracellular matrix to create a pro-tumorigenic microenvironment. These studies show that circadian disruptions regulate tumorigenesis via both protein-coding and non-protein-coding genes. Moreover, it can influence the tissue microenvironment to be either pro-tumorigenic or anti-tumorigenic by affecting cellular and non-cellular components. These findings underscore the need for further exploration in many areas.

PER2 is implicated in the modulation of DNA damage-responsive pathways, thereby mitigating tumor initiation [23]. Concurrently, another study identified that circadian disruption stemming from the loss of PER2 expression has been associated with a decline of MYC and CCNB1 repression in liver tissue [24]. Moreover, the absence of PER2 expression has been shown to elevate the levels of TNF-α and IL-6, thus augmenting tumor-related inflammation. In another research, PER2 downregulated by KMT2D induced a glycolytic vulnerability, resulting in the happen of lung cancer [25]. Circadian disruptions have also been linked to carcinogenesis in the liver through the upregulation of oncogenic gene transcription and inflammation. Conversely, there were different opinions in these results. Shaashua et al. [26] injected E0771 triple negative breast cancer cells and MC38 colon cancer cells to loss PER2 mice or wild mice. The tumor volume was significantly smaller in loss PER2 mice compared with wild mice. Thus, authors believed that PER2 was essential for tumorigenesis. Unfortunately, they did not compare the primary tumorigenesis ability between PER2-deficient mice and wild-type mice, which could provide higher quality evidence. There may be some confounding factors influencing this phenomenon. However, it is important to recognize that a specific clock gene may have different functions in various disease stages, organs and tissues.

Ohdo et al [27] elucidated that the loss of CLOCK inhibited the occurrence of chemically induced skin tumors. Specifically, CLOCK dysfunction reduced ATF4 repression, thereby elevating the transcription of P19ARF and the cellular senescence factor P16INK4A. Subsequently, RAS activation mediated by the EGF receptor was suppressed via the upregulated expression of P16INK4A, resisting skin carcinogenesis. Intriguingly, the upregulation of P16INK4A expression resulted in cellular senescence in mouse skin tissue, a phenomenon highlighting the involvement of circadian disruption in cell transformation. Balounová et al. [28] compared circadian rhythms and the cell cycle between 14-week-old and 33-week-old mice. Their findings suggested that aging moderately affected clock genes and significantly depressed cell cycle genes, leading to an uncoupling of the correlation between the clock and cell cycle in mice without tumors. As we know, senescence is characterized by cell cycle arrest [29, 30]. However, tumorigenesis predominantly clock genes rather than the uncoupling of circadian clocks and cell cycle, potentially explaining why aging occurs in the anti-tumor microenvironment. The evidence above suggests that the intricate interaction between the circadian clock and aging may offer a promising avenue for preventing tumorigenesis. In essence, aging is inevitable and often associated with various diseases, including cancer. Tumorigenesis could potentially be prevented or reduced by regulating the circadian rhythm appropriately.

**Figure 1.**
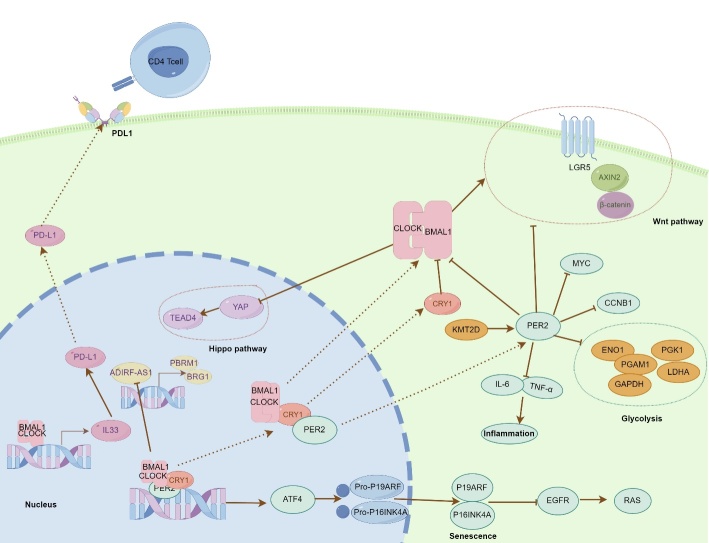
**Circadian rhythm affects carcinogenesis**. CLOCK/BMAL1 complex combines the E box promoter elements to regulate transcriptional levels of clock-controlled genes such as CRY1, PER2, and IL33. These clock genes and clock-controlled genes modulate carcinogenesis by regulating the relative pathways, inflammation, senescence, and immune cells in tumor microenvironment.

In another study, Schwartz et al. [31] found that chronic circadian disruption rather than pancreatic epithelial cell clock dysfunction promoted the pancreatic tumorigenesis in KRAS-mutant mice. In single-cell RNA sequencing results, fibroblast recruitment was observed in tumorigenic tissue and may correlate with carcinogenesis. This study may explain why night shift work or night light exposure may elevate the incidence of cancer [7, 32]. Thus, maintaining proper lifestyle habits may help prevent the occurrence of tumors, aligning with the requirements of aging. These findings collectively indicate that circadian rhythm modulates tumor initiation across various tissues through diverse mechanisms, such as life habits, immune environment modulation, inflammation induction, and senescence promotion (Fig. 1). This complex regulatory network warrants further investigation to potentially uncover strategies for tumor prevention.

## 3. Circadian rhythm and its impact on TME in cancer progression

### 3.1 Circadian rhythm and TME in cancer progression

#### 3.1.1 immune cells in TME

Circadian rhythm not only contributes to carcinogenesis but also influences the progression of cancer cells within the complex TME [33]. Within this background, diverse cellular constituents substantially affect tumor outcomes [34]. Immune cells, spanning both the innate and adaptive branches, possess the capability to kill cancer cells, forming the foundational principle of immunotherapy [35]. Patients could derive enhanced therapeutic benefits by judiciously selecting the optimal time for medication administration, aligning treatment with circadian immunological rhythm [36]. Macrophages play a key role in immune response. Of these, the interactions between circadian rhythm and aging can regulate immune homeostasis by affecting macrophages. Menon et al. [37] has proved that the attenuation in innate immune responses in aged tissue was caused by the downregulation of circadian gene expression in macrophages compared to young tissue macrophages. Mechanistically, the reduction of KLF4 in aging macrophages suppressed the transcription of circadian genes, leading to impaired innate immune responses. Among these, cancer-associated macrophages (CAMs), typically delineated into proinflammatory M1 and anti-inflammatory M2 phenotypes, play a pivotal role in cancer progression [38]. M1 polarization reduced circadian amplitudes and rhythmicities, while M2 polarization increased circadian periods in macrophages [39]. Furthermore, cancer cells and macrophages could influence each other's circadian rhythms. Research in melanoma has revealed that circadian rhythm altered the M1/M2 macrophage ratio in the TME, fostering a tumor promoting TME [40]. In another melanoma study, Lee et al. [41] reported that ablation of BMAL1 expression fostered an immunosuppressive subtype of CAMs that promoted melanoma cell proliferation by impairing mitochondrial metabolism and activating HIF-1A. This upregulation of HIF-1A subsequently cultivated an immunosuppressive TME characterized by reducing the infiltration of CD8+ T cells and NK cells. This study reminds us that CAMs not only directly affect cancer cells but also can forester a pro-tumor or anti-tumor TME by modulating immune cell infiltration.

In breast cancer, a study has categorized CAMs into anti-tumor and pro-tumor subtypes. The TME affected by circadian disruption exhibited a higher proportion of pro-tumor subtype CAMs infiltration, enhancing breast cancer cell proliferation [11]. Another study implicated that BMAL1 knockdown led to increased F4/80 CAMs infiltration in breast cancer, correlating with worse patient prognoses [42]. Further supporting these findings, lung cancer cells incubated with conditioned media from BMAL1-deficient CAMs showed augmented NLRP3 inflammasome activation, driving cancer cell proliferation, consisting to the effects that observed in breast cancer with BMAL1 knockdown [43]. These findings indicate that circadian disruption modulates cancer progression by influencing CAMs within the TME. Consequently, manipulation of circadian rhythm in CAMs presents a promising therapeutic avenue for cancer management. However, a serious problem appears in these studies. Many studies fail to distinguish and explore the specific functions of macrophage subtypes, limiting the value of these studies. Different subtypes of CAMs exhibited distinct functions in TME based on the regulation of circadian rhythm [39, 40]. Some subtypes of CAMs that fight against tumors may be hidden by other types that promote tumor growth, giving a misleading impression of CAMs overall. Future research needs to focus on investigating specific subtypes to gain a deeper understanding of the role of CAMs in the TME.

Moreover, the circadian regulator RORγ attenuated colorectal cell proliferation by increasing IL-17A production, extending T cell survival, and suppressing PD-1 expression in TME [44]. Concurrently, RORα modulated cholesterol metabolism through HDAC-mediated inhibition of the NF-κB pathway, affecting CD8+ T cell activity in the colon TME [45]. This evidence aligned with various studies highlighting the circadian regulation of CD8+ T cells and posited circadian rhythm as a promising way for anticancer therapy [46]. Within immune responses, elevated CLOCK expression was associated with diminished survival due to suppress NK cell infiltration. In terms of mechanism, TRIM35 expression leaded to the degradation of CLOCK protein, promoting NK cell infiltration in diffuse large B-cell lymphoma [47, 48]. Aging could affect immunity, including NK cell function [49, 50]. A study reported that long-term shift work would facilitate lung metastasis of B16F10 melanomas by promoting NK cell aging [51]. This inhibition of NK cell anti-tumor function was attributed to the downregulation of CD122 expression and induction of aging. The above results highlight the important role of aging in circadian rhythm affects TME again. The intricate interaction between the circadian clock and aging may offer a promising avenue for preventing tumorigenesis and inhibiting cancer progression. Furthermore, circadian dysfunction impacts both immune cell infiltration and their function, underscoring the importance of assessing both aspects in the TME. Collectively, circadian rhythm is integral in modulating both innate and adaptive immunity, indicating their potential as a kind of immunotherapy or to elevate the efficiency of immunotherapy.

#### 3.1.2 Other stromal cells and non-cell stromal components in TME

Many non-immune stromal cells and non-cellular components play significant roles in the TME, interacting with cancer cells to determine cancer progression. Of these, cancer-associated fibroblasts (CAFs) play a substantial role in orchestrating TME dynamics, promoting anti- or pro-tumor TME [52]. In the context of colorectal cancer, co-cultivation of cancer cells with CAFs, as opposed to fibroblasts from benign tumors, resulted in enhanced proliferative capacity [53]. Another colon study identified that co-culture with naive colon fibroblasts could decrease the growth ability of HCT116 compared to co-culture with CAFs via the modulation of circadian growth rhythm [54]. The decline of IL-6 secretion during naive colon fibroblasts progressed to CAFs was the reason why co-culture with CAFs had higher proliferation. Furthermore, endothelial cells within the TME significantly impact cancer patient prognosis and the efficacy of adjunctive therapies [55]. Circadian rhythm genes, such as BMAL1, can control endothelial cell cycle and thereby affect angiogenesis in development process and tumor progression [56]. For instance, in a glioma stem cell study, the CLOCK/BMAL1 complex within these cells was found to stimulate tumor angiogenesis within the glioma TME by modulating HIF1α through the transcriptional regulation of OLFML3 [57]. Building upon prior findings [58], the CLOCK/BMAL1 complex was observed to induce the expression of TBK1 in endothelial cells by secreting POSTN, thereby facilitating glioma TME angiogenesis. Notably, the pro-angiogenic effects were attenuated by SR9009, an inhibitor of CLOCK/BMAL1 complex transcription. One characteristic of solid tumors is their abundant vasculature, which provides sufficient nutrients for tumor cell proliferation. Circadian rhythm genes have been shown to regulate angiogenesis in the tumor microenvironment, suggesting a potential therapeutic approach for managing cancer progression. Additionally, targeting circadian rhythm genes may offer an alternative strategy to overcome resistance to anti-angiogenic drugs.

In the context of the glioma TME, glioma-associated microglial cells have garnered considerable attention among researchers [59]. Wang et al. [60] discovered that exosomes from M2 microglial cells, containing miR-7239-3p, repressed the expression of BMAL1, consequently promoting glioma cell proliferation. In another study, the CLOCK/BMAL1 complex within glioma stem cells demonstrated its capacity to upregulate the transcription of LGMN [58]. Mechanistically, the CLOCK-induced upregulation of OLFML3 enhanced LGMN transcription by modulating HIF1α in glioma stem cells. This elevated LGMN expression facilitated the recruitment of microglial cells to the TME by altering CD162 expression, resulting in increased glioma cell proliferation. Meanwhile, the recruited microglial cells fostered an immune-suppressive glioma TME, contributing to poorer patient survival. Microglial cells exhibited a key role in brain tumors. It can directly influence the progression of glioma and also culture a pro-tumor TME by regulating circadian rhythm. These results tell us that we need to focus on tissue specificity in various cancers. Each organ or tissue has its characteristics. For example, the gastrointestinal tract is mainly stimulated by meals throughout the day, while the lungs require continuous movement for respiration. Each organ or tissue has its own rhythmic characteristics. Thus, future research may pay attention to these characteristics to improve the understanding of each TME.

In addition to stromal cells, the dysfunction of PER2 within non-cell stromal components also curtailed tumor growth in breast and colon cancer [26]. In light of these findings, circadian rhythm exerts regulatory control over a multitude of stromal and non-cell stromal components, influencing cancer progression and offering promising avenues for cancer management.

#### 3.1.3 Cancer stem cells in TME

For cancer cells, cancer stem cells are pivotal in determining patient prognosis due to their influence on tumor evolution through modulation of TME and treatment responses [61, 62]. Hence, the impact of circadian disruption on these cells has become a focal point of investigation. In breast cancer, stem cells derived from a disrupted circadian environment exhibit enhanced tumorigenicity when introduced into normal mice compared to those from a regular circadian milieu [11]. The BMAL1/CLOCK complex, essential for maintaining circadian rhythm, is fundamentally necessary for the proliferation of glioma stem cells, with its inhibition curtailing their growth [63]. In addition to promote the growth of stem cells in glioma, the BMAL1/CLOCK complex could also recruit immunosuppressive microglia to the TME, thereby establishing conditions associated with poorer prognoses [64]. Chen et al. [65] revealed in prostate cancer that suppression of PER3 escalated BMAL1 expression, which in turn activated the WNT/β-catenin pathway in TME, augmenting cancer stem cell stemness. These observations underscore the necessity of circadian integrity for cancer stem cell maintenance and suggest that circadian manipulation may synergize with immunotherapy by altering the tumor immune microenvironment.

### 3.2 Circadian rhythm and TME in the metastasis of cancers

**Figure 2.**
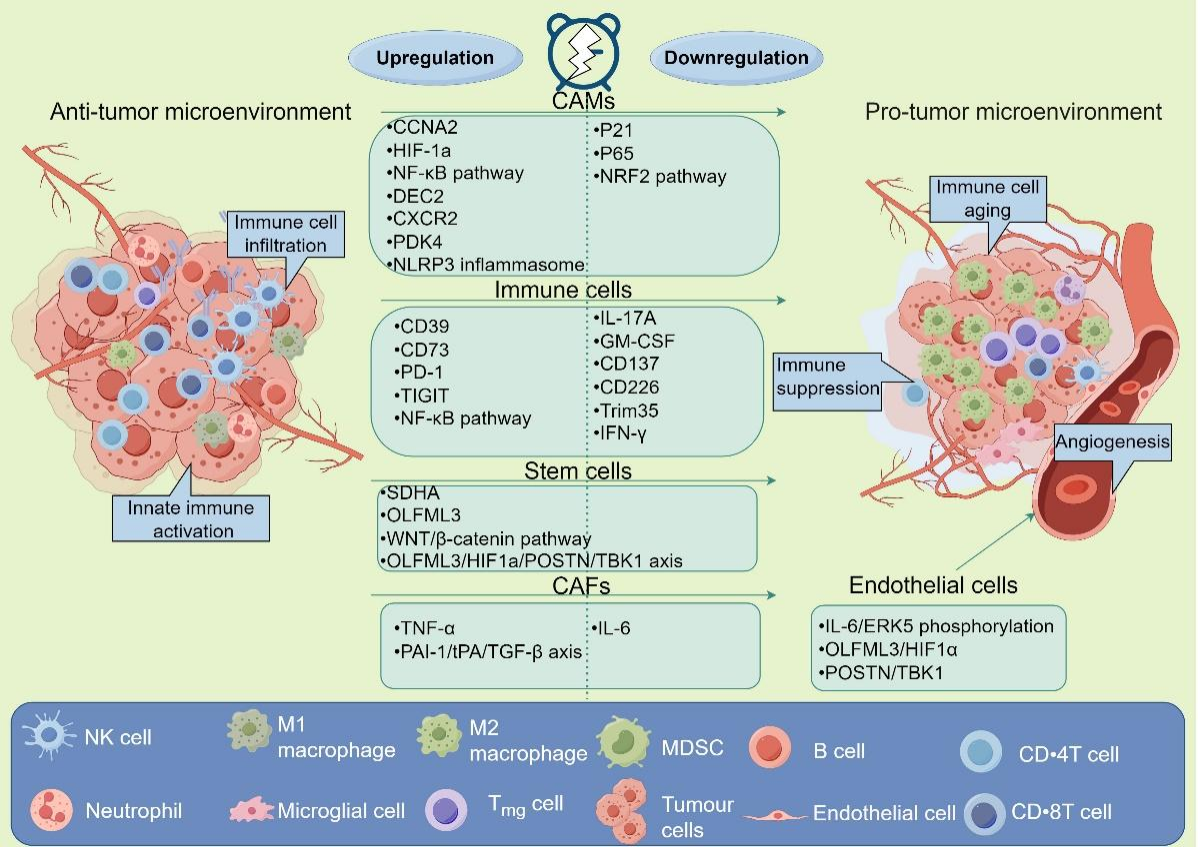
**Circadian rhythm involves the whole process of cancer**. Circadian rhythm changes the situation between anti-tumor and pro-tumor microenvironment by regulating the expression of clock genes in cancer and stromal cells. The clock-controlled genes and their productions modulate tumor microenvironment biology, including cancer stem cells, cancer-associated macrophages (CAMs), cancer-associated fibroblasts (CAFs), immune cells, endothelial cells, and so on.

The occurrence of distant metastasis typically signifies the advanced stage of cancer in patients. Inhibiting the metastasis of cancer cells is crucial for extending the life expectancy of cancer patients [66]. In terms of CAMs, Lee et al. [43] reported that lung cancer cells cultured in conditioned medium from BMAL1-deficient CAMs exhibited an enhanced epithelial-mesenchymal transition through NLRP3 inflammasome activation, which could be attenuated by a specific inhibitor of the NLRP3 inflammasome, MCC950. Similar in colorectal cancer, overexpression BMAL1 in cancer cell lines (HCT116 and SW620) could promote cancer cell distant metastasis [67]. Specifically, BMAL1 expression elevated cancer cell secreting RAB27A which induced the migration of endothelial cells and cancer cells. In a colon cancer, it was observed that the loss of PER2 expression was associated with a lower ratio of CAFs infiltration, resulting in a reduced metastasis rate [26]. In this context, Cao et al. [68]reported that the loss of BMAL1 expression promoted the transition of CAFs into alpha smooth muscle actin-positive myofibroblasts (myoCAFs), consequently increasing the metastasis of colorectal cancer. Mechanistically, circadian disruption caused by mutating BMAL1 reduced the expression of PAI-1 in the TME. Subsequently, tumor metastatic ability was heightened due to the increased infiltration of myoCAFs activated through the TGF-β pathway, facilitated by the downregulation of PAI-1. In agreement with these findings, another study reported that the circadian gene Rev-erbα regulated the transition of fibroblasts into myoCAFs in human lung fibroblasts induced by TGFβ1, as evidenced by changes in αSMA and COL1A1 expression [69]. Both studies identified Rev-erbα as a regulator of the fibroblast-to-myoCAF transition, thereby influencing disease progression. The fibroblast-to-myoCAF transition is a core process in circadian rhythm control of cancer metastasis, underscoring the importance of identifying stromal cell subtypes. Moreover, disrupting stromal cell transitions to pro-tumor subtypes through circadian rhythm regulation may effectively manage cancer. For instance, inhibiting fibroblast-to-myoCAF transition could halt distant metastasis in colorectal cancer [68]. This suggestion is further supported by CAMs transition and polarization [39]. Manipulating circadian rhythm shows promise in regulating the direction of stromal cell transition. This approach holds potential for managing cancer metastasis and tumor progression. Taken together, these findings underscore the role of circadian rhythm in regulating various cell types, impacting cancer metastasis (Fig. 2).

## 4. Circadian rhythm and TME in the management of cancers

Drug resistance is a pervasive issue in clinical practice, arising from various mechanisms, including gene mutations, abnormal metabolism and so on [70, 71]. Therefore, it is imperative to comprehensively investigate the specific mechanisms of drug resistance and develop strategies to overcome this challenge. In this regard, circadian rhythm has shown the capacity to reverse drug resistance by modulating the expression of relevant genes [13, 72]. In a study focused on melanoma, DEC2-induced circadian rhythm was found to regulate the transcription of the PD-1 receptor in CAMs by downregulating P65. During the light phase, upregulation of DEC2 resulted in low PD-1 expression, leading to reduced efficacy of BMS-1, a PD-1/PD-L1 inhibitor. Conversely, during the dark phase, downregulation of DEC2 induced high PD-1 expression, enhancing the efficacy of BMS-1 [73]. Additionally, RORγ agonists have demonstrated potential as immunotherapeutic agents [44]. These agonists exerted control over cancer cell growth by promoting the infiltration of T17 cells, which, in turn, upregulated the expression of IL-17A while downregulating PD-L1 expression. As previously mentioned, glioma-associated microglia are regulated by the CLOCK-HIF1a-LGMN-CD162 pathway [58]. Further investigations have revealed that downregulating the CLOCK/BMAL1 complex or employing LGMN/CD162 inhibitors in microglia significantly enhanced CD8+ T cell activity and suppressed PD-L1 expression, potentially synergizing with anti-PD-1 therapy. These findings suggest that circadian rhythm represents a promising avenue for immunotherapy, potentially enhancing the effectiveness of current immunotherapeutic approaches.

**Figure 3.**
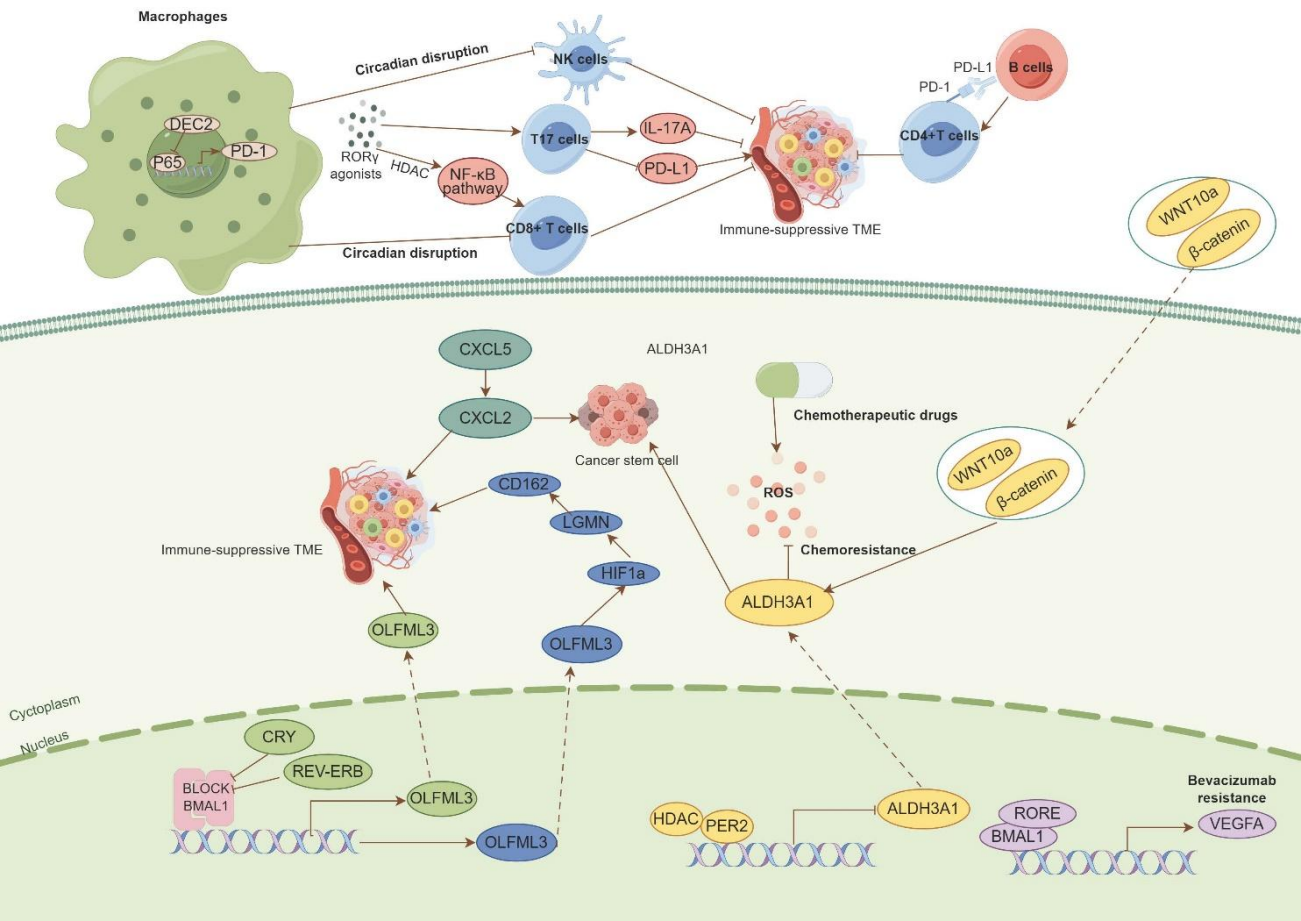
**Circadian rhythm participates in the cancer treatment**. Circadian rhythm modulates the immune environment by the clock and clock-controlled genes which regulate the infiltration and function of immune cells, exhibiting synergistic potential with immunotherapy and offering a promising avenue for innovative immunotherapeutic strategies. Meanwhile, the clock and clock-controlled genes also regulate the chemoresistance through modulate drug-induced accumulation of reactive oxygen species (ROS), cancer stem cells, immune tumor microenvironment (TME).

Chemoradiotherapy has been a mainstay of clinical practice for many years; however, overcoming chemoradiotherapy resistance remains a formidable challenge [74]. Ohdo et al. [75] reported that cancer cells originating from fibroblasts with loss of PER2 expression exhibited resistance to chemotherapy when compared to wild-type cancer cells. Further experiments revealed that the loss of PER2 expression failed to inhibit the transcription of ALDH3A1, which played a role in suppressing drug-induced accumulation of reactive oxygen species. Another study also demonstrated that ALDH3A1-induced circadian rhythm could regulate the population of ALDH-positive triple-negative breast cancer cells [76]. In line with these findings, circadian disruption was observed in colon cells co-cultured with CAFs compared to those co-cultured with normal fibroblasts, which also led to chemoresistance [53]. In the context of radiotherapy, increasing the expression of PER1/2 in the TME significantly enhanced the sensitivity of glioma cancer cells to radiation [77]. These results suggest that circadian rhythm may offer a promising approach to overcoming chemoradiotherapy resistance by modulating the TME. The above evidence paves the way for the application of chronotherapy in current cancer treatments. The specific mechanism of the above results has been exhibited in Figure 3.

## 5. Chronotherapy perspectives in TME

Cancer treatments, including surgery, chemotherapy, and immunotherapy, will be reduced effectiveness, and caused side effects by disrupting circadian rhythm [78-80]. Researchers aim to enhance anti-tumor therapies’ efficacy and minimize adverse effects by modulating circadian rhythm. For instance, in a glioblastoma study, TMZ exhibited highest anti-tumor ability when BMAL1 at daily maximum expression in vitro and in vivo [81]. Lehr et al. [82] proposed a personalized chronomodulated 5-Fluorouracil and enhanced efficacy based on dihydropyrimidine dehydrogenase activity. Similarly, circadian rhythm is involved in the immune regulation of TME. Scheiermann et al. [83] have identified that dendritic cells CD8+ T cells through the BMAL1/CD80 axis, inducing circadian anti-tumor to control melanoma volume in mice. In terms of immunotherapy, esophagus cancer patients received nivolumab before 13:00 were significantly associated with better progression-free survival compared to those receiving it later [84]. Based on these findings, researchers have conducted many clinical studies on chronotherapy. Clinical applications of chronotherapy mainly involve comparing chronomodulated drug with conventional schedules, using circadian rhythm-modulating drugs in combination with anticancer agents, modifying lifestyle habits, and so on. In terms of chronochemotherapy, most current studies believed it could decrease the side effects of chemotherapy [85]. A randomized controlled trial (RCT) come from European reported that metastatic colorectal cancer patients received chronomodulated chemotherapy shown lower side effect rate than those undergoing conventional chemotherapy [86]. Another RCT come from China also reported that chronoradiotherapy was significantly associated with less side effects for cancer patients [14]. In terms of immunotherapy, a trail come from America proved that chronoimmunotherapy could bring better immune responses and less toxicity in patients with advanced melanoma [80]. There are many chronotherapy RCTs ongoing, such as NCT04864405, NCT04735939, and so on. In combination chronotherapy, the addition of melatonin to chemotherapy resulted in improved survival outcomes compared to chemotherapy alone in cancer patients [87]. Circadian disruption induced by lifestyle habits significantly affected the prognosis of cancer patients [88]. Thus, doctors try to change the environment of cancer patients. A RCT come from America indicated that chronotype-tailored light therapy could significantly improve the life quality of breast cancer patients receiving adjunct therapies [89]. Many studies support the positive effect of chronotherapy in cancer management, while some researchers doubt about this opinion [90]. Thus, higher-quality studies are still needed to demonstrate the effectiveness of chronotherapy. Additionally, consensus and standardized protocols for the clinical application of chronotherapy have yet to be established, which should be the focus of future efforts. Furthermore, there would be significant costs associated with medical resources. If patients are only treated in the morning, medical equipment and staff will remain unused in the afternoon. Hospitals also need to expand existing medical resources to meet the demand for treatment during the same time period, which poses a significant healthcare burden for developing countries. For patients, receiving treatment only at fixed times can lead to overcrowding and concerns about treatment availability. There are various solutions to address this issue, all of which just should be taken into account during the development of chronotherapy.

## 6. Conclusion and perspective

Circadian rhythm represents a fundamental adaptation of human biology to environmental cues. Numerous studies have underscored the significant role of circadian rhythm in cancer [57]. Researchers have begun to explore chrono-pharmacological strategies for the management of cancer patients in clinical practice [14, 91]. Therefore, it is imperative to review and summarize the involvement of circadian rhythm in the TME to promote the application of chrono-pharmacological approaches in cancer management. Drawing from the aforementioned findings, we have delineated the specific roles of circadian rhythm in various phases of cancer, encompassing initiation, progression, and metastasis. Circadian rhythm exerts a profound influence on the entire spectrum of cancer processes in TME. Additionally, they demonstrate synergistic potential with current anti-tumor treatments, offering a promising avenue for innovative chronotherapeutic strategies.

In TME, the interactions between aging and circadian rhythm have significant effects in cancer development and progression. Aging is an unavoidable process of human and has different characteristics compared with young stage [28, 92]. The incidence rate of cancer usually increases with aging [93, 94]. Meanwhile, the world is entering an era of aging population [95]. Understanding the roles of aging and circadian rhythms in cancer and leveraging their principles for effective prevention and treatment of tumors are promising and crucial tasks ahead. Furthermore, life habits affect treatment efficiency and cancer initiation and progression [96-98]. These results suggest that we need to culture proper life habits based on circadian rhythm, especially for aged people. For example, the frequency, timing, and types of meals may influence the human circadian clock through stimulation or interaction with the resident microbiota [99]. Of these, oral and gut microbiota also play a significant role in the efficiency of cancer treatments [100, 101]. Thus, cultivating good lifestyle habits and circadian rhythms may help the body effectively resist tumor occurrence and progression. Furthermore, there are many new drugs to control cancer by influencing circadian rhythm [102]. Drug delivery systems also appear as an anti-tumor way [103, 104]. To accommodate the human's circadian rhythm, researchers are developing circadian drug delivery systems. These systems aim to optimize drug delivery by releasing medication at specific times according to the body's circadian rhythm, potentially enhancing efficacy, minimizing side effects, and improving patient compliance [105]. Despite the challenges such as patient-specific considerations, circadian drug delivery systems remain a promising direction worth pursuing. Moreover, natural drugs have applied in clinical work many years [106, 107]. Recently, some natural products exhibited anti-tumor ability by affecting circadian rhythm [108, 109]. For example, 1,25-dihydroxyvitamin D could prevent skin carcinogenesis by affecting circadian rhythm [110]. Resveratrol could play a circadian clock modulator to manage cancer [111]. The role of the circadian rhythm in the TME is complex, closely associated with multiple pathways and phenotypes. Clarifying the specific mechanisms of the circadian rhythm in the TME is crucial for addressing existing drug resistance and developing new therapies. Fortunately, modern techniques such as single-cell sequencing, organoid cultures, spatial proteomics, and artificial intelligence can be employed to investigate the specific mechanisms of the circadian clock in the TME. Addressing these and related questions in future research endeavors will further facilitate the integration of circadian rhythm into everyday life and cancer management. While some TME-based chronotherapy has shown promising clinical outcomes in clinical trials, further exploration is needed to understand the role and mechanisms of the circadian rhythm in the TME.
